# Supplementary material for: Tracking matricellular protein SPARC in extracellular vesicles as a non-destructive method to evaluate lipid-based antifibrotic treatments
Source: Commun Biol. 2022 Oct 30;5:1155. doi: 10.1038/s42003-022-04123-z (PMC9618575; doi:10.1038/s42003-022-04123-z)
Supplement: Supplementary file 2 — Supplementary Information [file 42003_2022_4123_MOESM2_ESM.pdf]

Supplementary Information

Tracking matricellular protein SPARC in extracellular vesicles as a non-destructive method to evaluate lipid-based antifibrotic treatments

Cristina Zivko[1,2,3], Kathrin Fuhrmann[3], Gregor Fuhrmann[3,4*], Paola Luciani[1,2*]

[1] *Institute of Pharmacy, Friedrich Schiller University of Jena, Jena, Germany*
[2] *Department of Chemistry, Biochemistry and Pharmaceutical Sciences, University of Bern, Bern, Switzerland*
[3] *Helmholtz Institute for Pharmaceutical Research Saarland, Department of Pharmacy, Saarland University, Saarbrücken, Germany*
[4] *Current address: Department of Biology, Friedrich-Alexander-University Erlangen, Erlangen, Germany*

*Email to: gregor.fuhrmann@fau.de; paola.luciani@unibe.ch

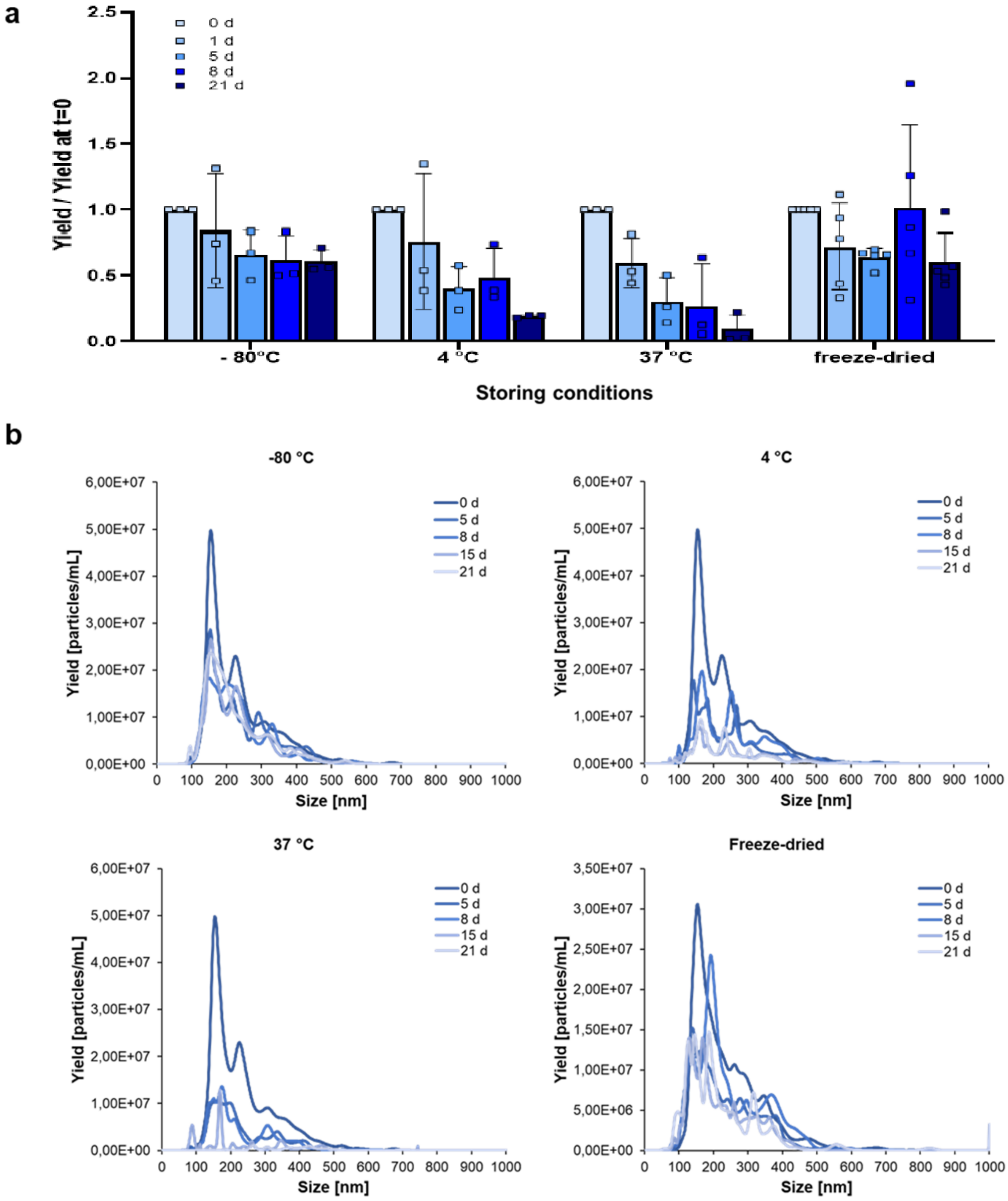

Fig. S1 | a, EV yield upon mid-term storage under different conditions for up to 21 d (mean ± SD, n = 3-5, software: NTA v3.2). **b**, EV size distribution profiles upon mid-term storage under different conditions for up to 21 d.

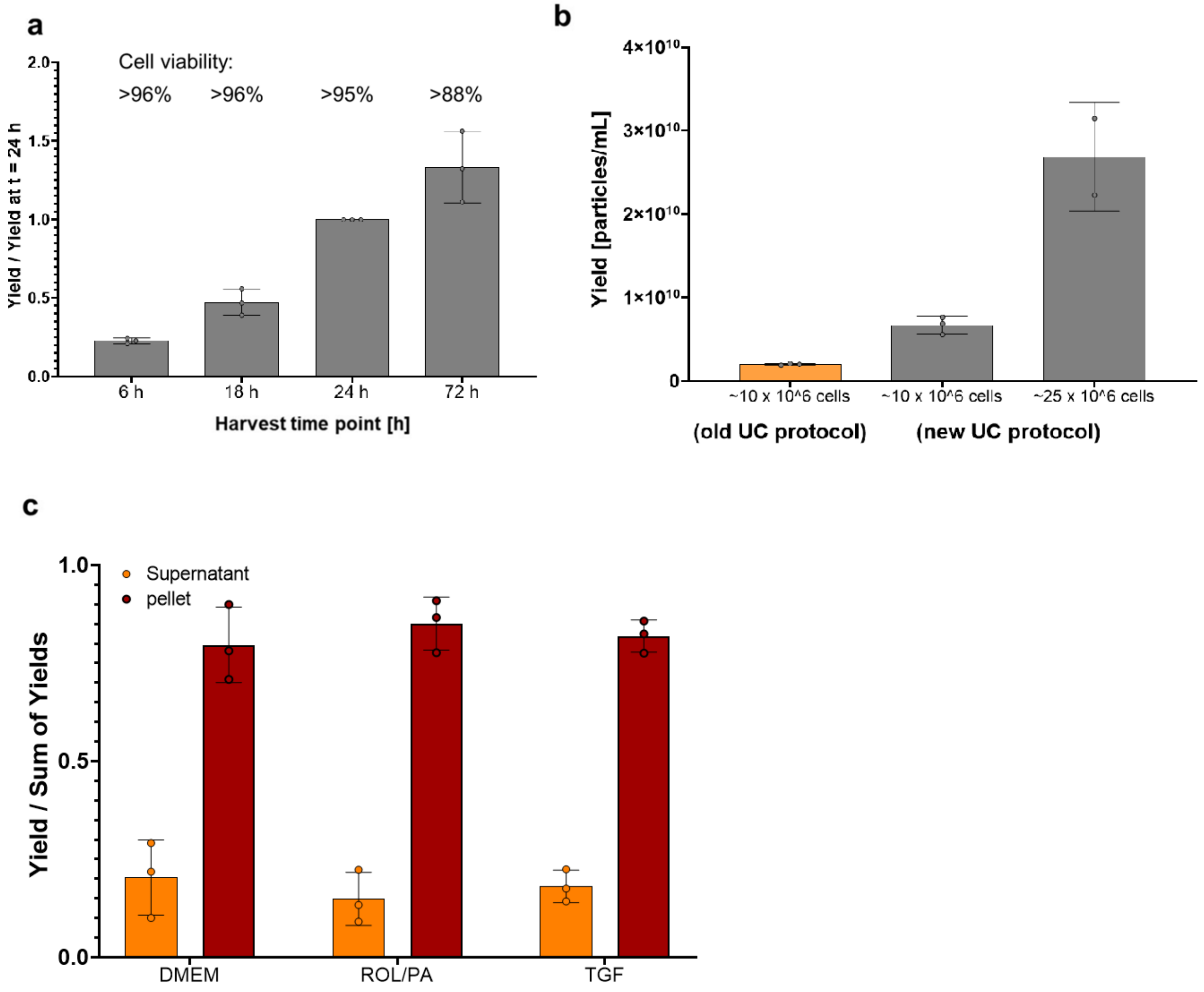

Fig. S2 | a,b, EV yield at different harvesting time points (**a**) and upon changing the UC protocol (**b**). For the old protocol, CCMs were centrifuged at 300 x g for 5 min at 4 °C, the supernatant was moved into a new tube and centrifuged again (10’000 x g, 20 min, 4 °C). The pelleted cell debris was discarded and the supernatant was ultracentrifuged (100’000 x g, 1 h 30 min, 4 °C). The three centrifugation steps were updated for the new UC protocols as described in the methods: 300 x g for 3 min, 9’000 x g for 30 min, 120’000 x g for 2 h 30 min. **c**, Comparison between the pelleted particles’ yield and the particles’ yield in the supernatant after UC. Mean ± SD, n = 2-3, software: NTA v3.2.

Table S1 | Average cell count, cell viability, EV yield, EV size and EV zeta potential values after isolation of EVs from differently treated LX-2 (mean ± SD, n = 3).

	**Cell count (cell viability%)**	**EV yield [particle number]**	**EV Size [nm]**	**EV Zeta Potential [mV]**
**DMEM (untreated)**	$10.9 \pm 1.1 \times 10^6$ (97.2 ± 3.1)	$8.43 \pm 2.4 \times 10^9$	178 ± 87	-39.9 ± 2.2
**ROL/PA (quiescent-like)**	$9.3 \pm 1.2 \times 10^6$ (96.5 ± 2.5)	$9.91 \pm 3.9 \times 10^9$	185 ± 93	-34.1 ± 2.5
**TGF (perpetuated)**	$11.7 \pm 1.4 \times 10^6$ (96.2 ± 3.1)	$9.07 \pm 3.5 \times 10^9$	182 ± 93	-36.6 ± 2.7

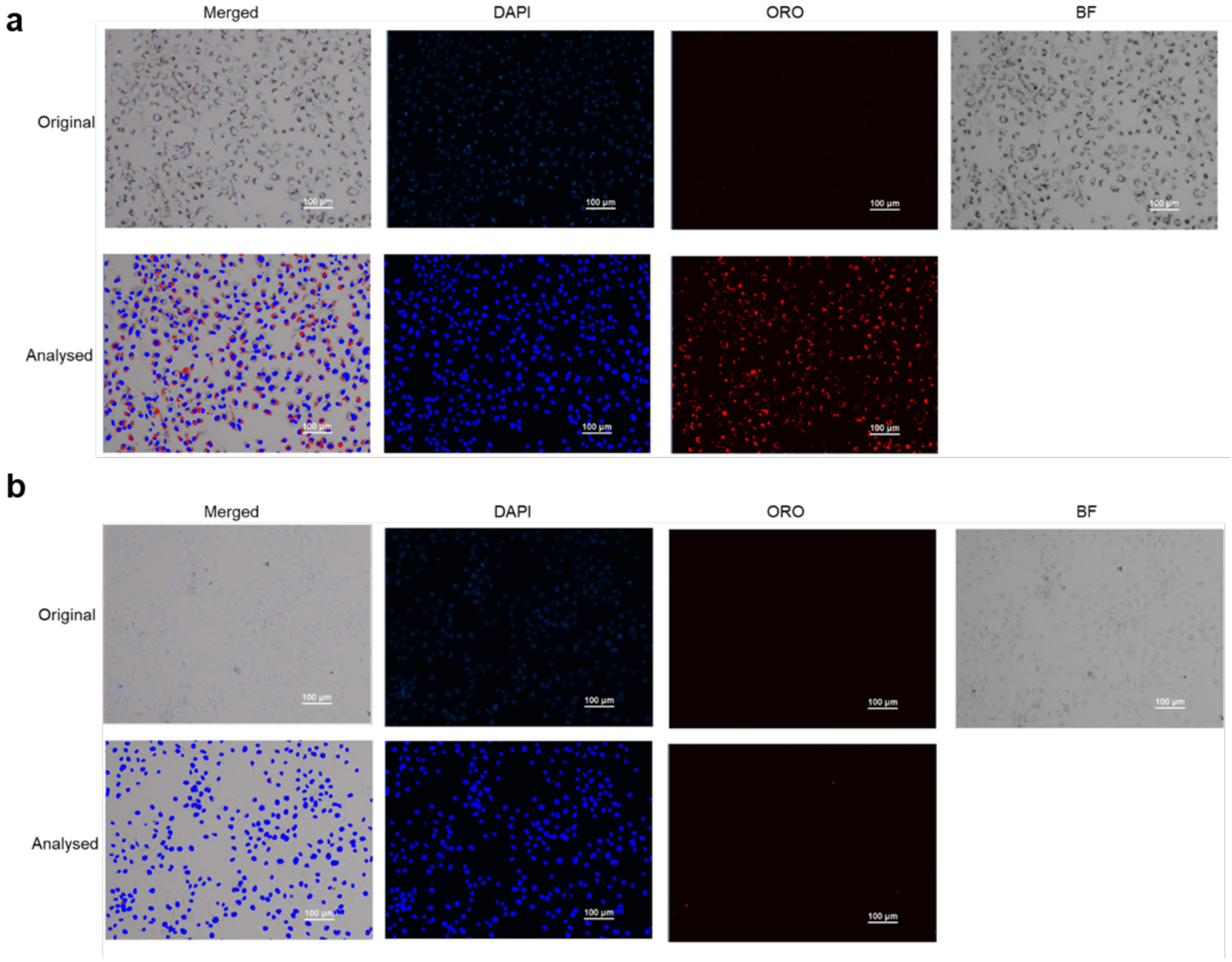

Fig. S3 | a,b, Thresholding compromise for the analysis of fluorescence microscopy images. a, Representative microscopy image of ROL/PA-treated cells split into acquisition channels. The upper row shows the original pictures taken on the same spot, whereas the bottom row shows the same pictures after thresholding. It is readily seen (especially after merging), how the software is able to detect all single DAPI-stained nuclei, and almost all red areas corresponding to cytoplasmic lipid droplets (i.e, almost perfect detection of true positives). **b**, Representative microscopy image TGF-treated cells split into acquisition channels. The upper row shows the original pictures taken on the same spot, whereas the bottom row shows the same pictures after thresholding. It is readily seen (especially after merging), how the software is able to detect all single DAPI-stained nuclei, and almost no red areas corresponding to cytoplasmic lipid droplets (i.e., almost perfect avoidance of false positives). All pictures of LX-2 cells were acquired with the same settings and analysed using the same thresholding.

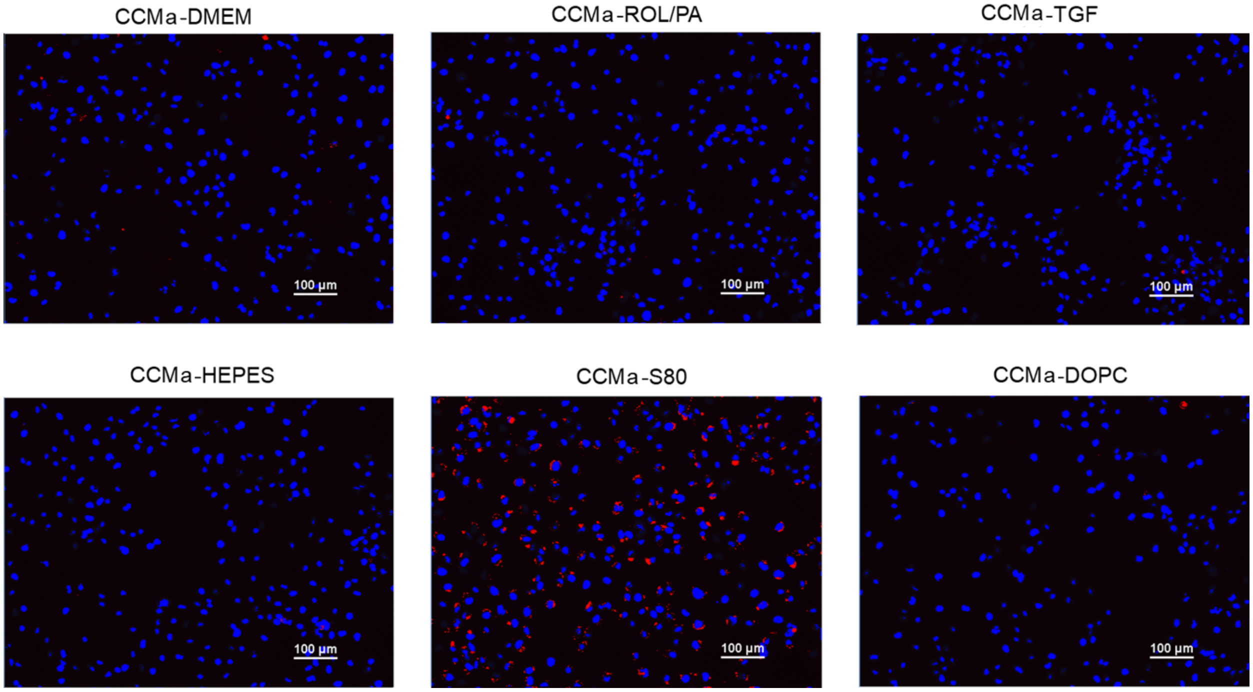

Fig. S4 | Representative images of ORO staining in fluorescence (seen as red areas; nuclei stained with blue DAPI) of differently treated cells after thresholding. LX-2 cells were treated with CCMa-EVs from previously treated HSCs.

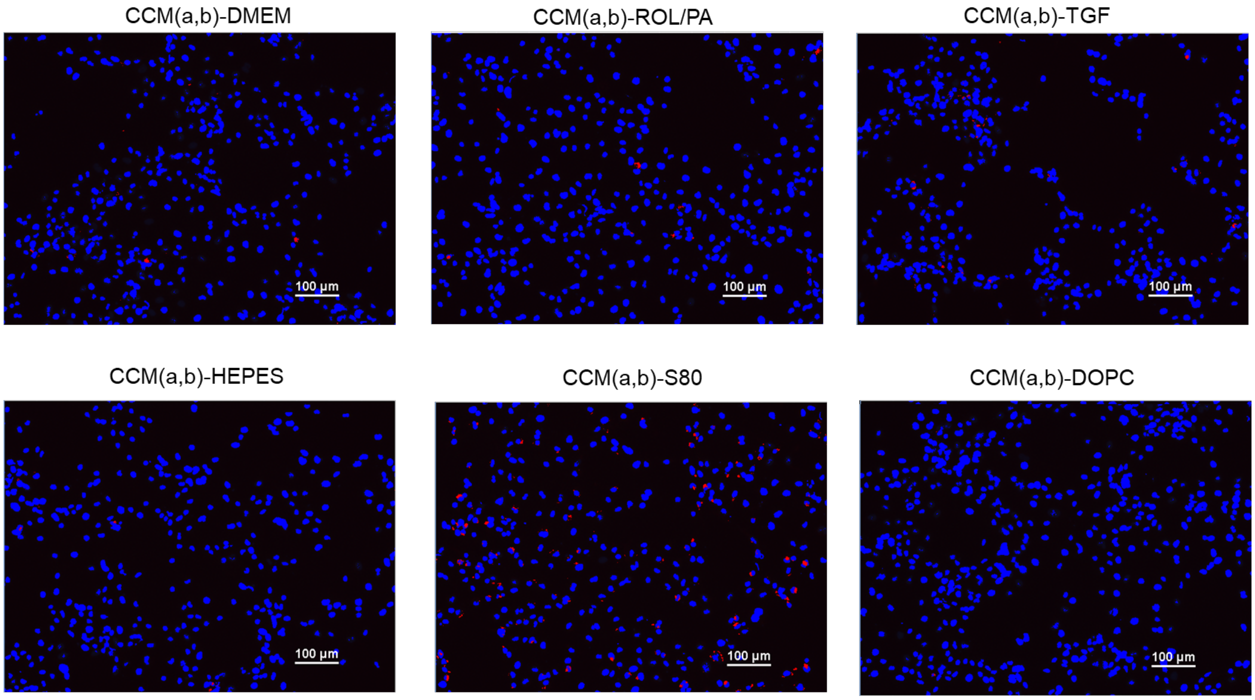

Fig. S5 | Representative images of ORO staining in fluorescence (seen as red areas; nuclei stained with blue DAPI) of differently treated cells after thresholding. LX-2 cells were treated with CCMa-EVs from previously treated HSCs for 24 h, washed with PBS then treated for 24 h with CCMb-EVs from previously treated LX-2.

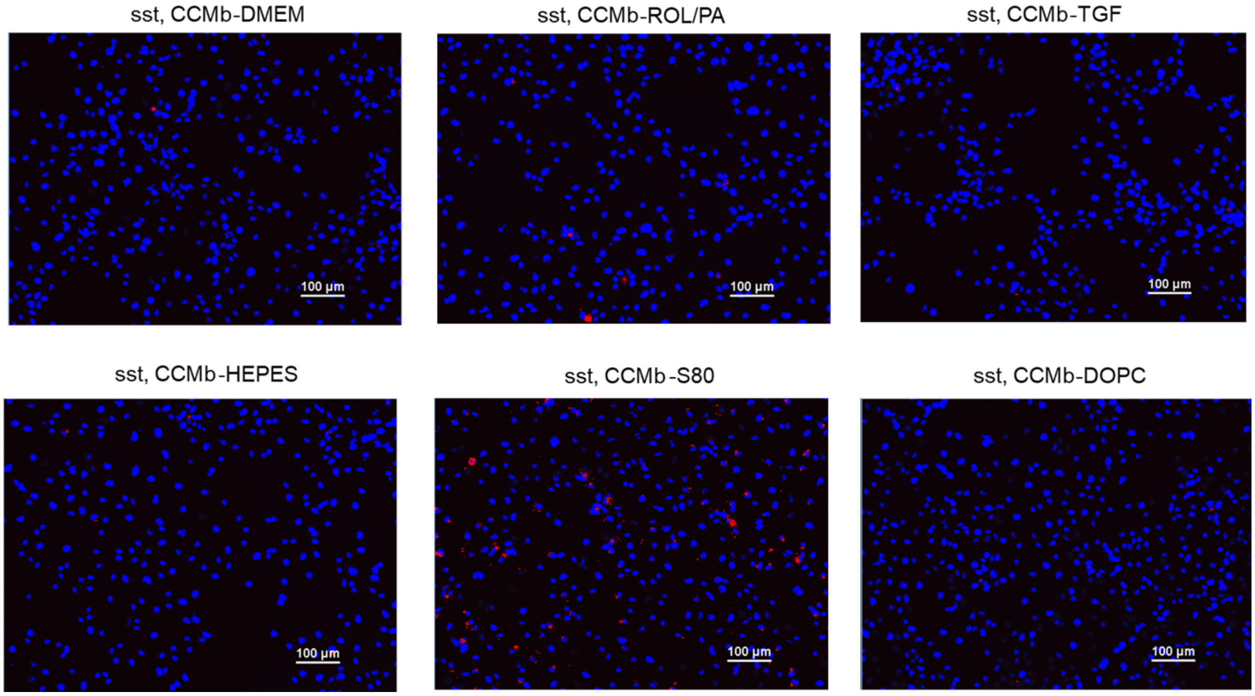

Fig. S6 | Representative images of ORO staining in fluorescence (seen as red areas; nuclei stained with blue DAPI) of differently treated cells after thresholding. LX-2 cells were treated with serum-free DMEM for 24 h (serum starved, sst), washed with PBS then treated for 24 h with CCMb-EVs from previously treated LX-2.

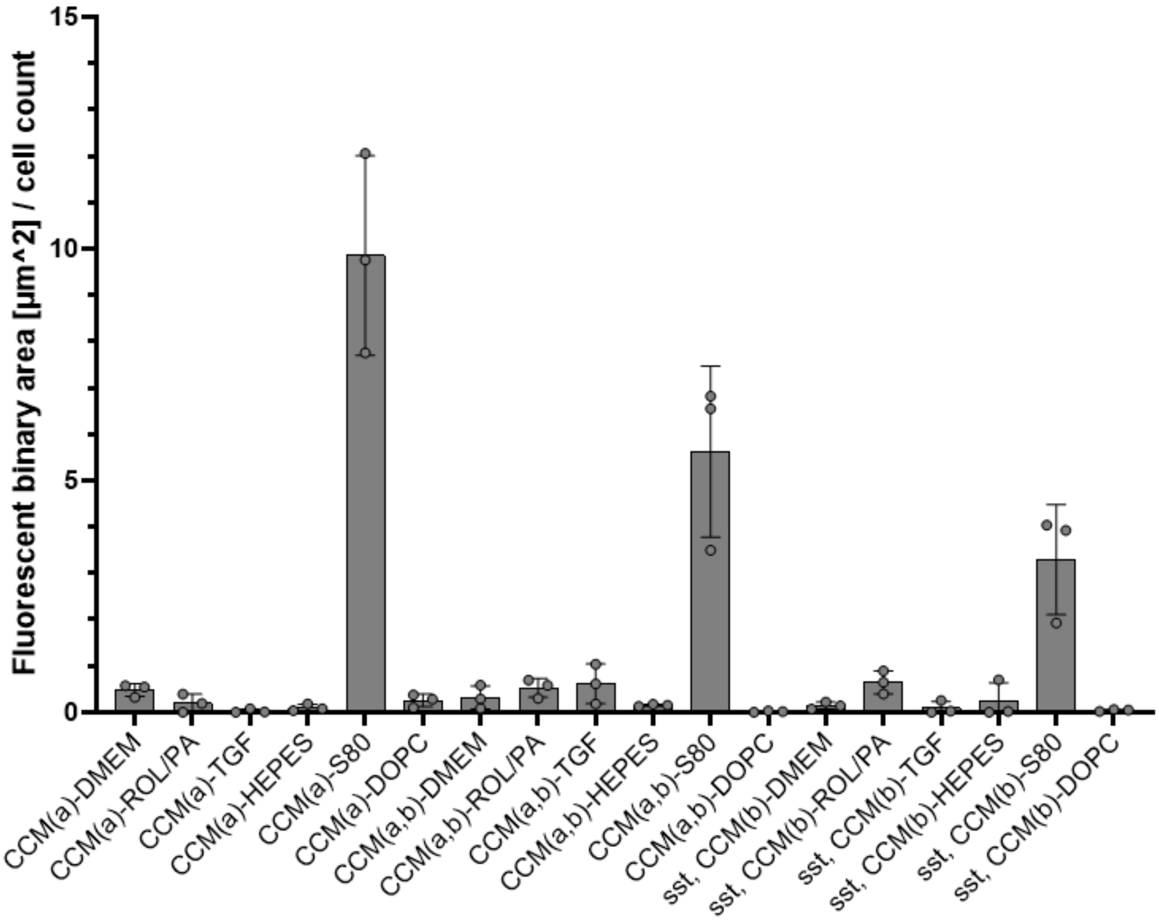

Fig. S7 | Quantitative analysis of stained lipid droplets, whereby the fluorescent area (correlating to a quiescent-like status) was normalized to cell count. Results from direct treatment are compared with CCMb-EVs in Fig. 2.

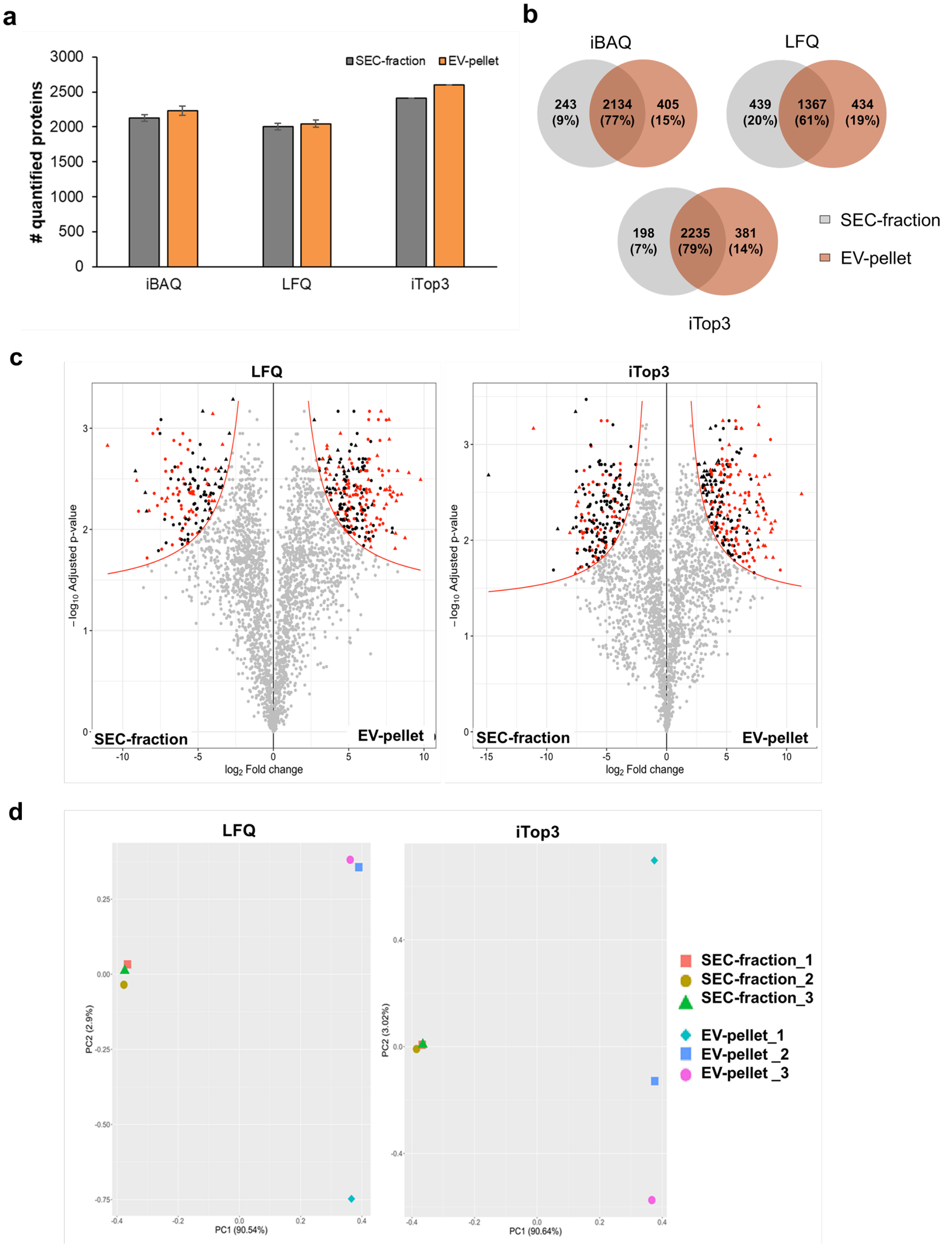

Fig. S8 | a,b Extraction of EV-associated proteins from EV-pellet and SEC-purified fractions led to the consistent quantification of over 2’000 proteins (**a**), with a considerable overlap between EV-pellets and SEC-purified EVs (**b**). These observations were similar for all quantification approaches, i.e., with iBAQ (intensity-based, absolute quantification, useful in some context but rarely used), iTop3 (based on the detected intensity of the top three peptides) and LFQ (label-free quantification) as shown with Venn diagrams (b). The latter two methods are frequently used on their own, but when both quantification methods are adopted, comparing the results from LFQ and iTop3 can be used to confirm findings from one with the findings from the other. **c**, Volcano plots offer a closer look into the comparisons of proteomic

profiling results. A significance curve was calculated based on a minimal log2 fold change of 1 and a maximum adjusted p-value of 0.05, and is shown as a red line: everything below that curve is not considered to be significantly different between SEC-purified EVs and EV-pellet samples. There are however differences between samples, and data points are marked in red when there is agreement between LFQ and iTop3 evaluations, which would give them even more credence. **d**, Principal component analysis (PCA) shows similarity degrees between biologically independent triplicates undergoing the same sample preparation in LFQ and iTOP3 analyses. Looking at the grouping of replicates by PCA, we could show that SEC-purified samples are more reproducibly similar to each other than proteomic profiles of EV-pellets, resulting in much clearer grouping. This does not come as a surprise given the additional purification step, but it is now supported by more validated data.

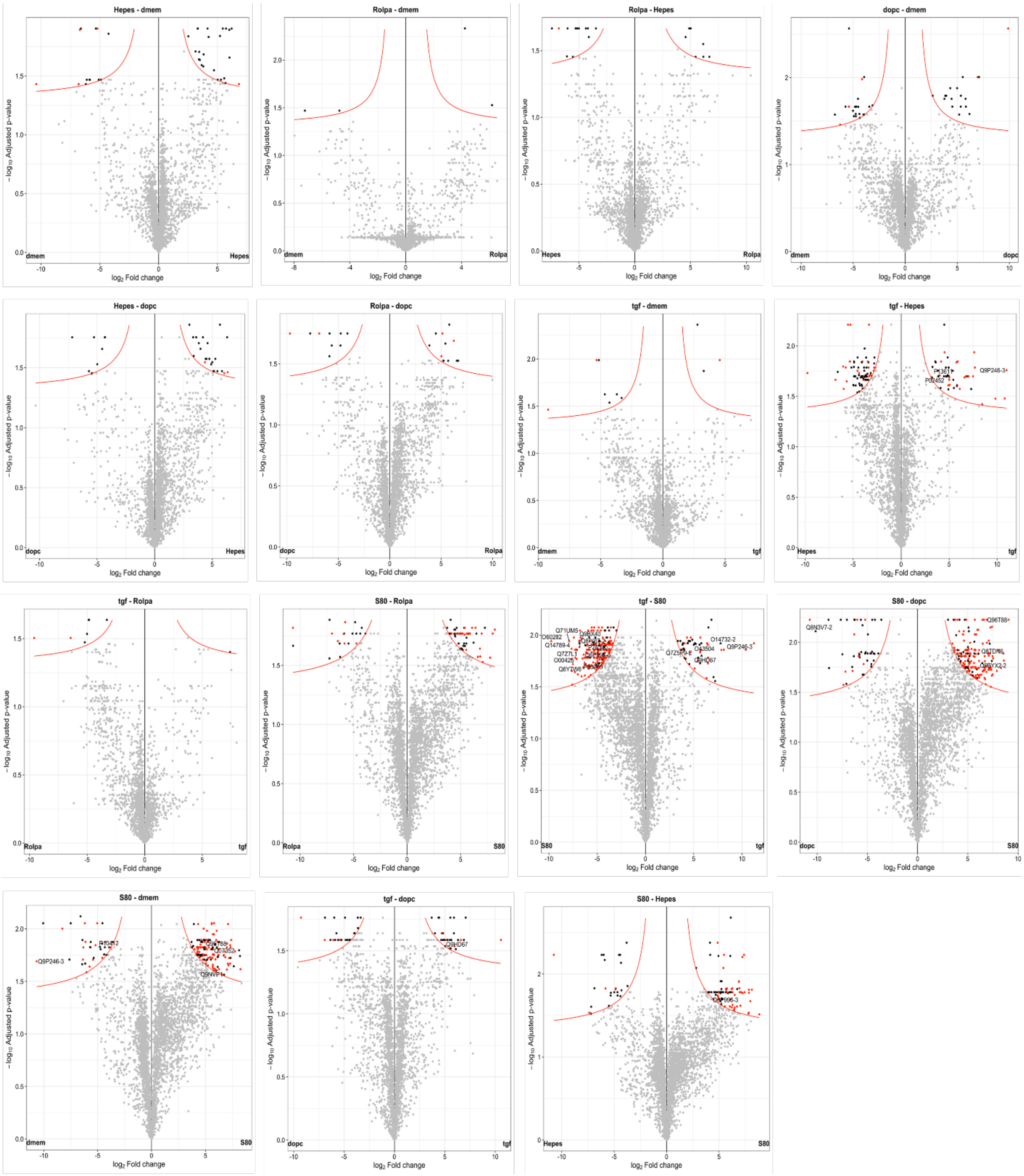

Fig. S9 | Volcano plots with direct comparisons of treatment groups based on EV-proteins from SEC-purified samples (using LFQ).

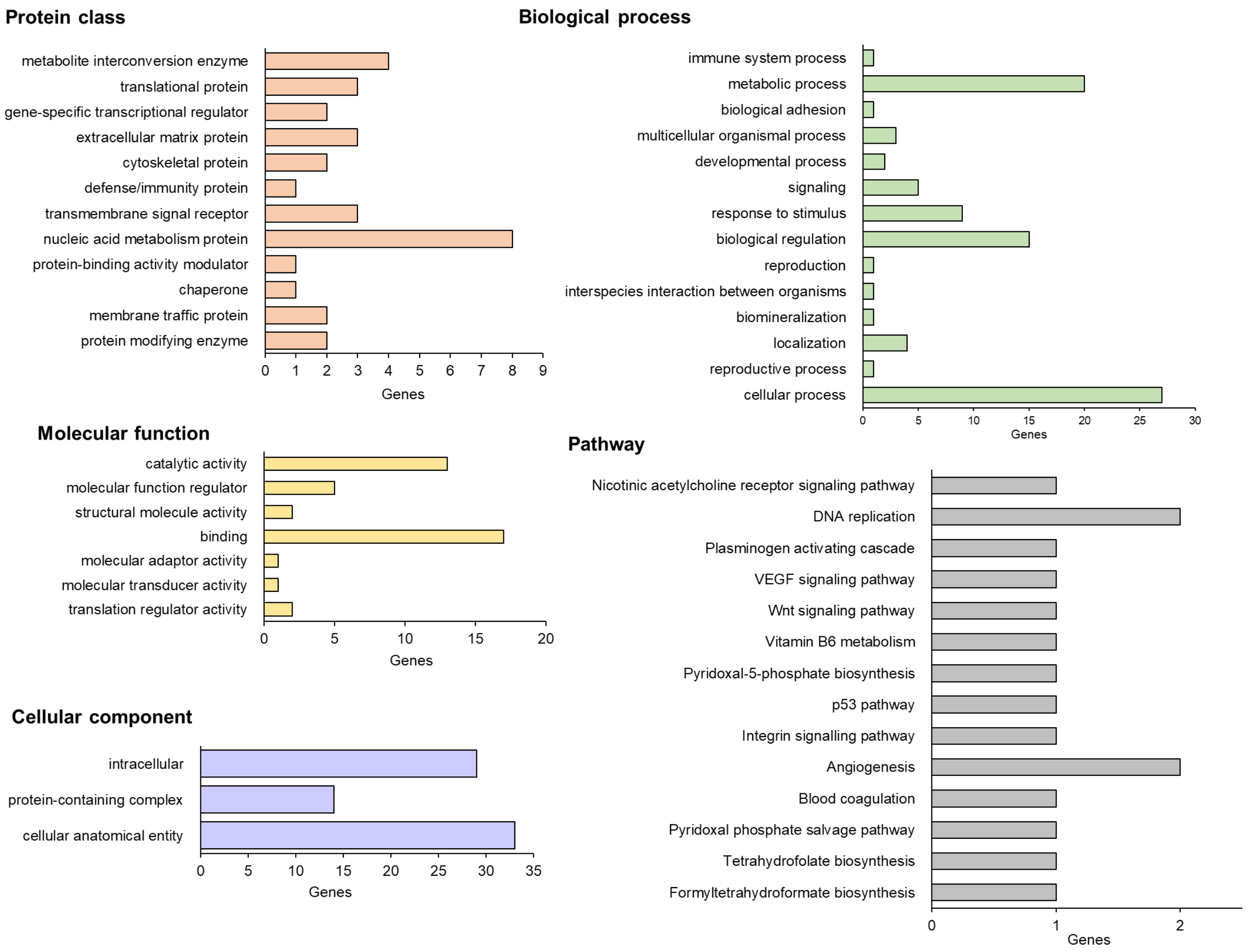

Fig. S10 | Classification of the 44 proteins comprising the treatment-discriminating panel using the PANTHER platform.

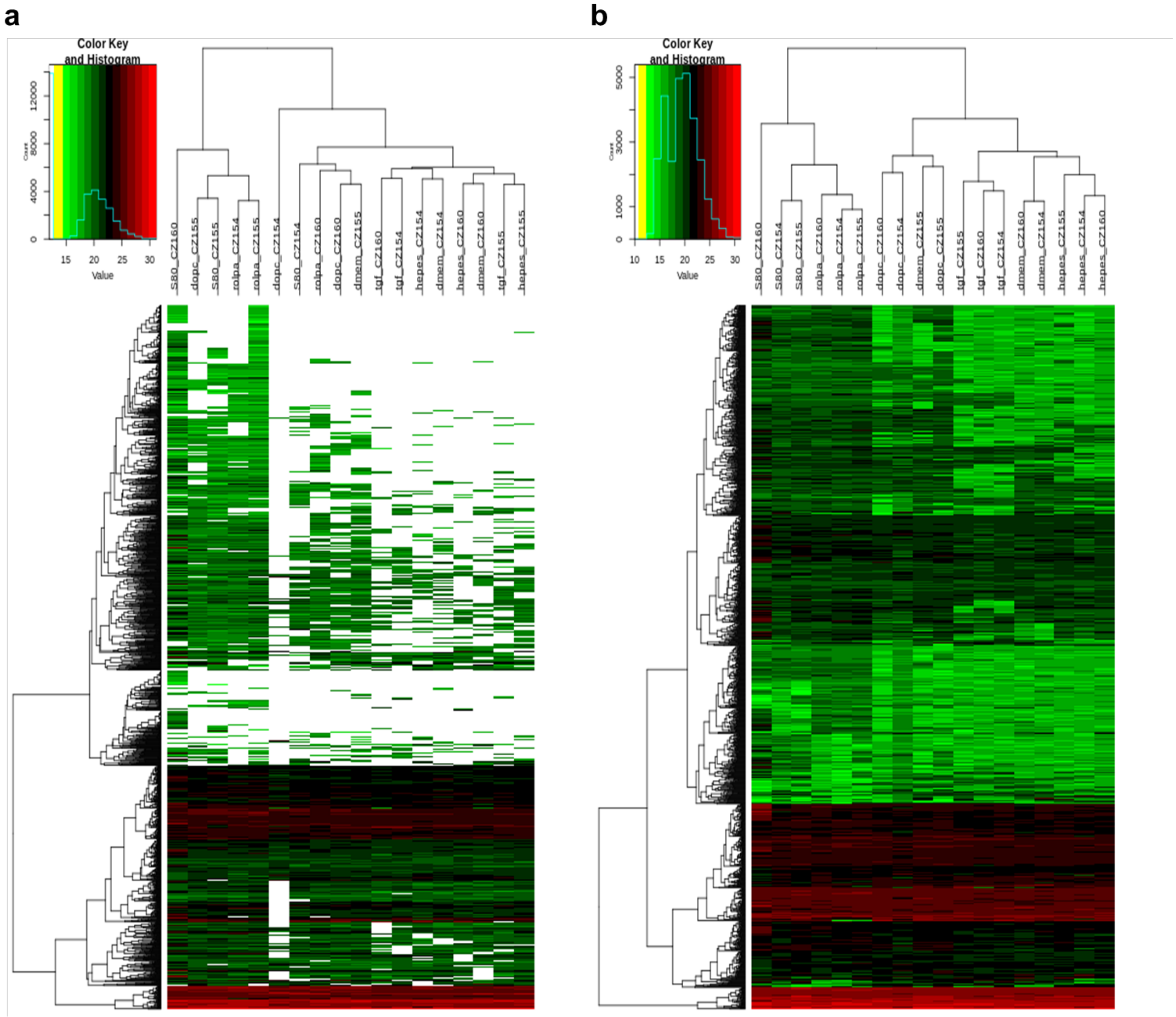

Fig. S11 | Hierarchical clustering with of LFQ before (**a**) and after imputation (**b**) for the combined AF4-peaks. Per sample, imputation values were drawn from a gaussian distribution of width 0.3x sample standard deviation centered at the sample distribution mean minus 2.5x sample standard deviation. This is a left-censored method and is done if there is at most 1 non-zero values in the group for a protein. Any remaining missing values are imputed by the Maximum Likelihood Estimation (MLE) method.

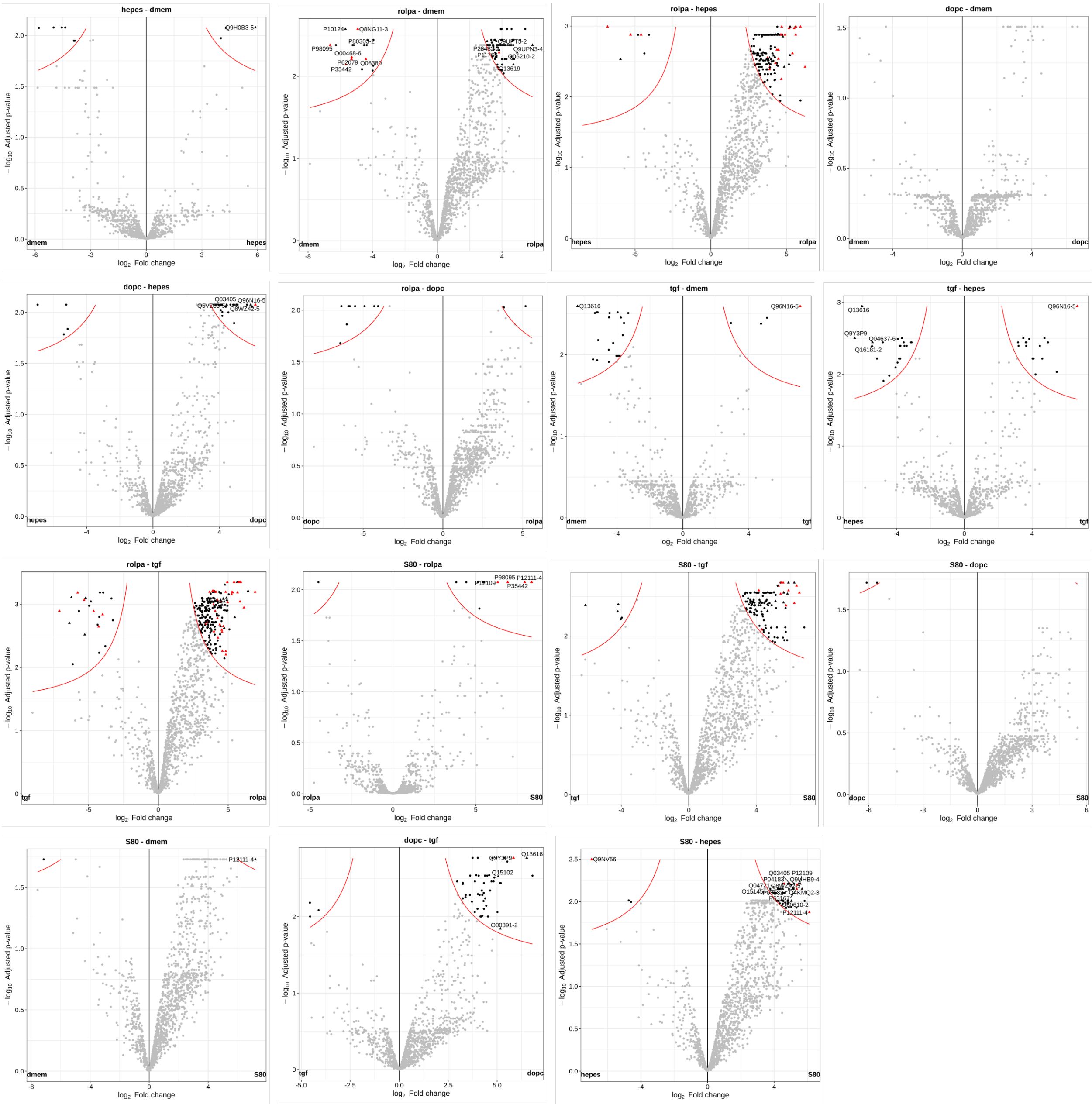

Fig. S12 | Volcano plots with direct comparisons of treatment groups based on EV-proteins from AF4-purified samples, peaks combined (LFQ).

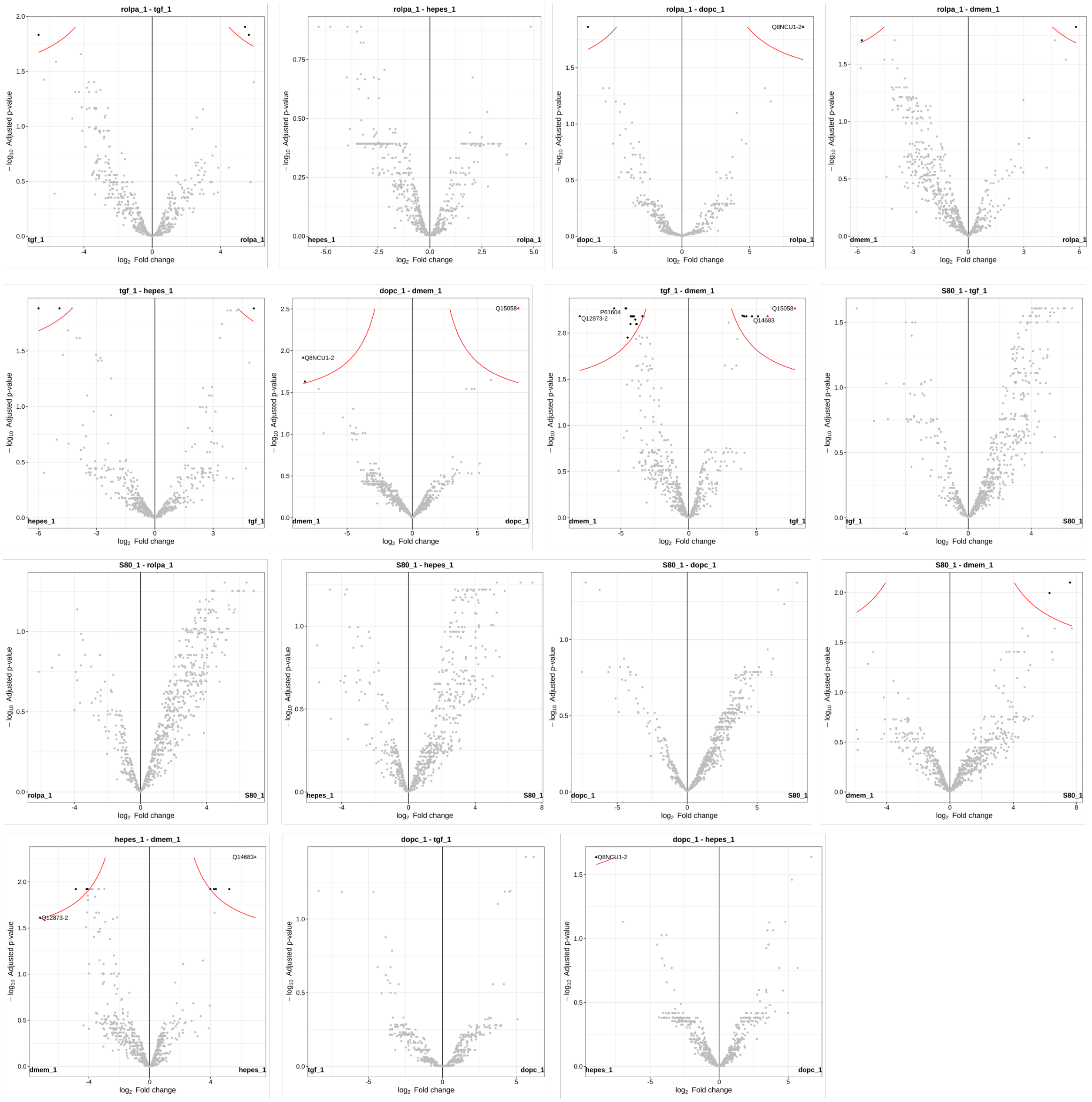

Fig. S13 | Volcano plots with direct comparisons of treatment groups based on EV-proteins from AF4-purified samples, peak 1 (LFQ).

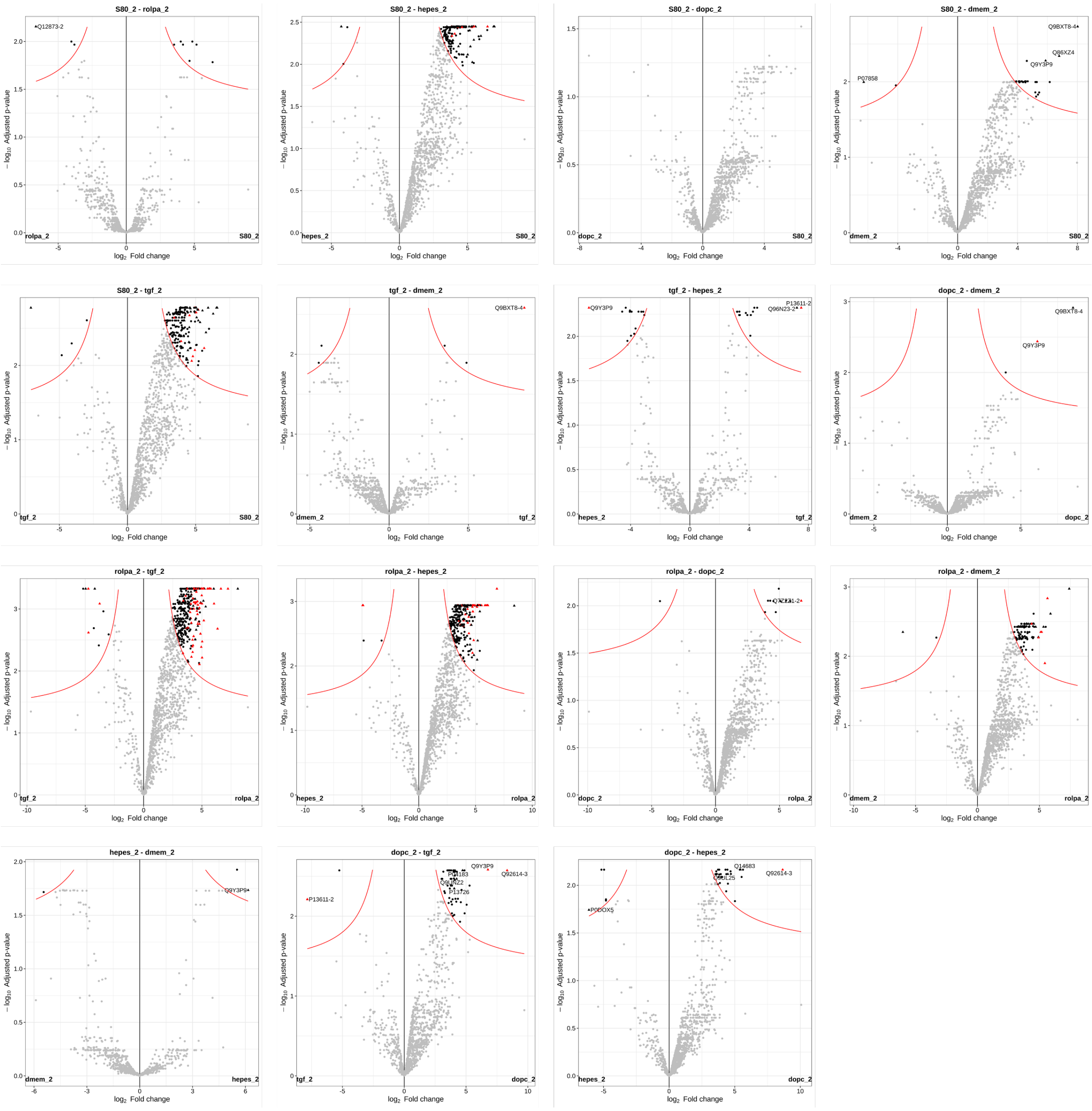

Fig. S14 | Volcano plots with direct comparisons of treatment groups based on EV-proteins from AF4-purified samples, peak 2 (LFQ).

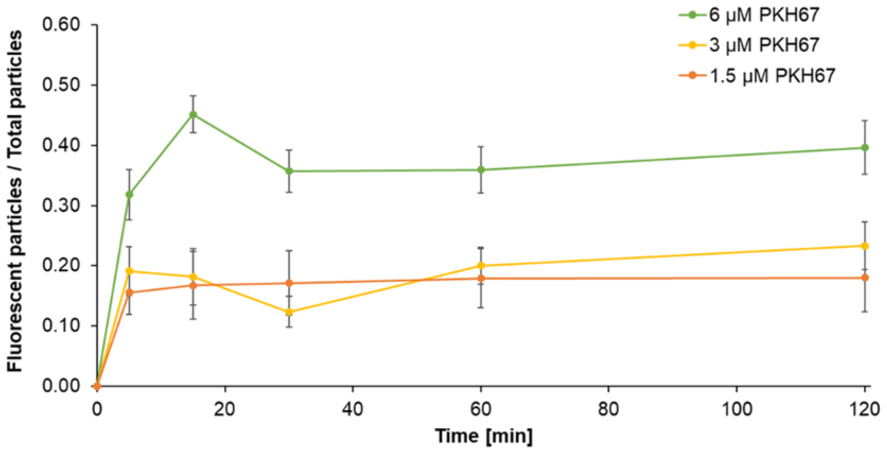

Fig. S15 | SEC-purified EVs from untreated LX-2 stained with different concentrations of unspecific membrane dye PKH67 for different incubation times (mean ± SD, n = 3, software: ZetaView 8.05.05 SP2).

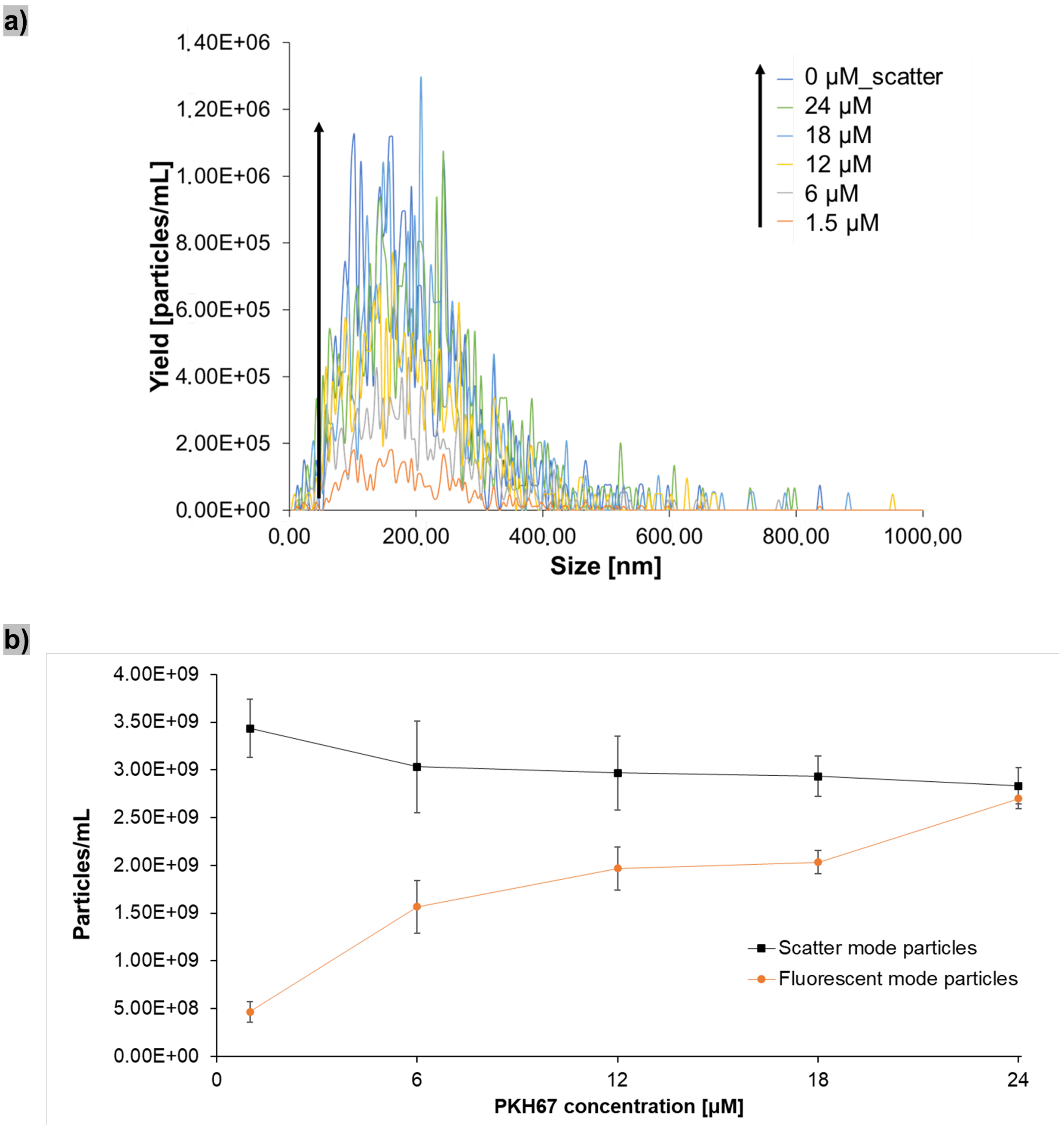

Fig. S16 | Representative size distribution profiles of particles measured in fluorescence mode upon addition of increasing amounts of PKH67 (a), and average particle count in scatter and fluorescent mode (b) (mean ± SD, n = 3, software: ZetaView 8.05.05 SP2).

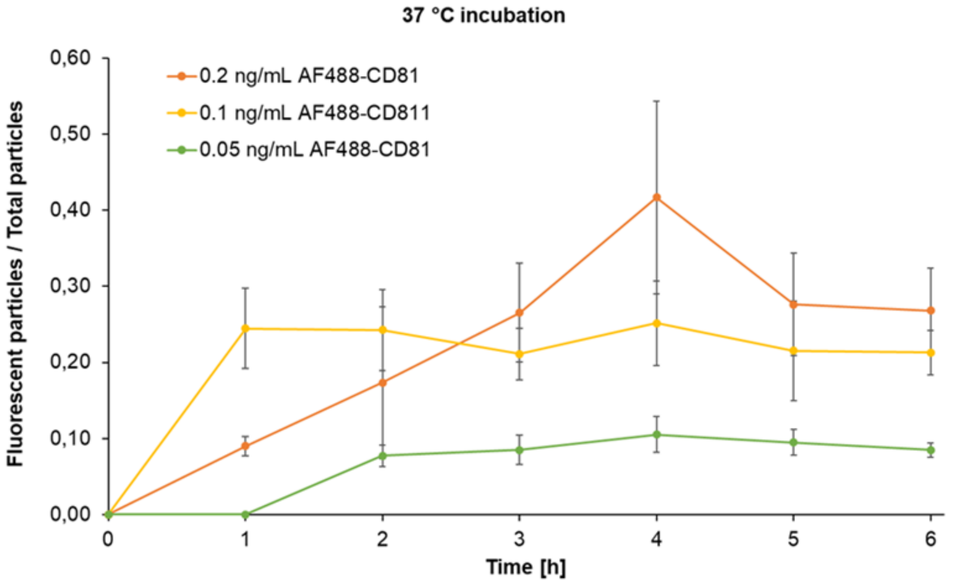

Fig. S17 | SEC-purified EVs from untreated cells incubation with varying amounts of AF488-CD81 and for different times at 24 °C (**a**) and at 37 °C (**b**) (mean ± SD, n = 3, software: ZetaView 8.05.05 SP2).

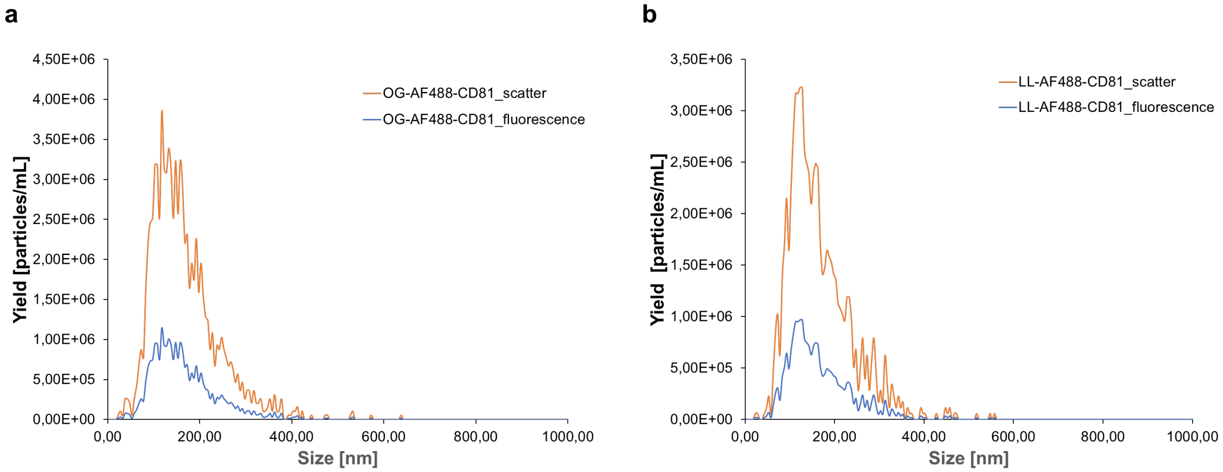

Fig. S18 | Representative size distribution profiles of particles measured in scatter and fluorescence mode upon addition of AF488-CD81, using the original (OG, purchased already AF488-conjugated) probe (**a**), or using the Lightning-Link® (LL) labelled probe we conjugated (**b**).

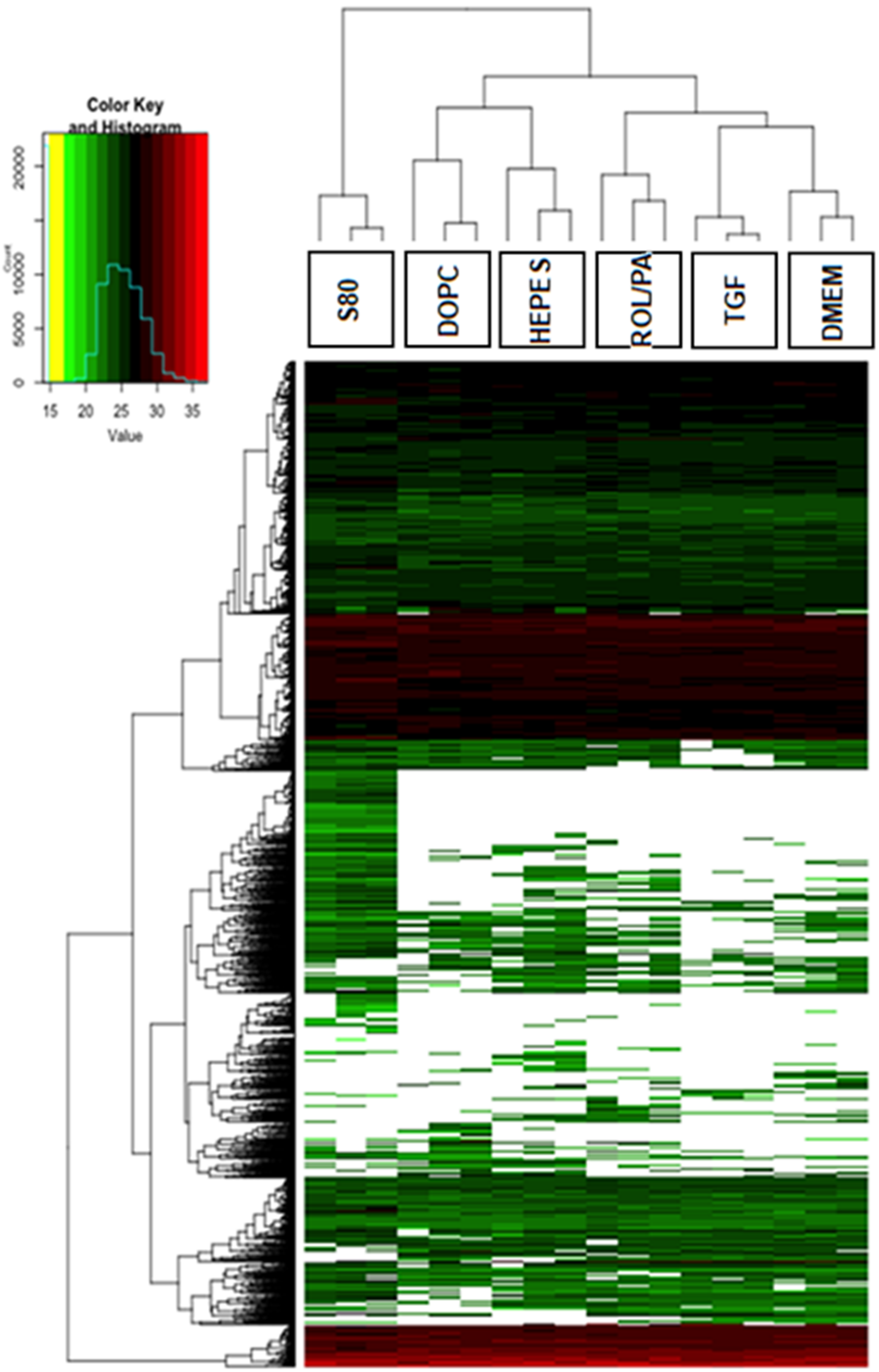

Fig. S19 | Hierarchal clustering from Fig. 3b shown on its own for better visualization.

Table S2 | Listed out proteins from Fig. 3f.

ROLPA AND S80 over TGF		TGF over ROLPA AND S80
IFIT3_HUMAN	BRE1A_HUMAN	SRCRL_HUMAN
IKKB_HUMAN	TBC9B_HUMAN	SPRC_HUMAN
LSM1_HUMAN	MAP1S_HUMAN	NCS1_HUMAN
RPAC1_HUMAN	TTI2_HUMAN	TICN1_HUMAN
KPRB_HUMAN	RS27L_HUMAN	
KIF5C_HUMAN	PEG10_HUMAN	
RNH2A_HUMAN	CCAR1_HUMAN	
RGS20_HUMAN	P20D2_HUMAN	
PLPHP_HUMAN	TTC9C_HUMAN	
HAUS5_HUMAN	ZN579_HUMAN	
HS74L_HUMAN	NEK7_HUMAN	
DPOLA_HUMAN	NELFB_HUMAN	
IFIT1_HUMAN	EXOS8_HUMAN	
IDE_HUMAN	GCP3_HUMAN	
NLTP_HUMAN	SEH1_HUMAN	
MK03_HUMAN	CYFP2_HUMAN	
GRAN_HUMAN	CHAP1_HUMAN	
3MG_HUMAN	ERGI2_HUMAN	
GPC1_HUMAN	RANB9_HUMAN	
RFC5_HUMAN	PNKP_HUMAN	
PCP_HUMAN	FYCO1_HUMAN	
EI2BB_HUMAN	PELO_HUMAN	
PLCD1_HUMAN	NUF2_HUMAN	
STA5B_HUMAN	ADNP_HUMAN	
MAZ_HUMAN	HAUS4_HUMAN	
LSM6_HUMAN	CNO10_HUMAN	
SMAD3_HUMAN	ELP3_HUMAN	
FOXK1_HUMAN	SPC25_HUMAN	
PGBM_HUMAN	MUC5B_HUMAN	
GOGA3_HUMAN	MOV10_HUMAN	
STX5_HUMAN	KI13B_HUMAN	
KCC2G_HUMAN	RHG35_HUMAN	
KCC1A_HUMAN	RBM12_HUMAN	
SF3A2_HUMAN	PANK4_HUMAN	
TKFC_HUMAN	SPN90_HUMAN	
VP26B_HUMAN	VPS18_HUMAN	
PDCD4_HUMAN	STAR9_HUMAN	
NU188_HUMAN	COMD3_HUMAN	
MTCL1_HUMAN	TBK1_HUMAN	
