# Supplementary material for: Tracking matricellular protein SPARC in extracellular vesicles as a non-destructive method to evaluate lipid-based antifibrotic treatments
Source: Commun Biol. 2022 Oct 30;5:1155. doi: 10.1038/s42003-022-04123-z (PMC9618575; doi:10.1038/s42003-022-04123-z)
Supplement: Supplementary file 3 — Description of Additional Supplementary Files [file 42003_2022_4123_MOESM3_ESM.pdf]

Description of Additional Supplementary Files

File name: Supplementary Data 1
Description: List of proteins from the proteomics study.

File name: Supplementary Data 2
Description: The single point source data behind the graphs in the paper.
