# Supplementary material for: Tracking matricellular protein SPARC in extracellular vesicles as a non-destructive method to evaluate lipid-based antifibrotic treatments
Source: Commun Biol. 2022 Oct 30;5:1155. doi: 10.1038/s42003-022-04123-z (PMC9618575; doi:10.1038/s42003-022-04123-z)
Supplement: Supplementary file 6 — Reporting summary [file 42003_2022_4123_MOESM6_ESM.pdf]

nature portfolio

Corresponding author(s): Gregor Furhmann/Paola Luciani

Last updated by author(s): Oct 15, 2022

Reporting Summary

Nature Portfolio wishes to improve the reproducibility of the work that we publish. This form provides structure for consistency and transparency in reporting. For further information on Nature Portfolio policies, see our Editorial Policies and the Editorial Policy Checklist.

Statistics

For all statistical analyses, confirm that the following items are present in the figure legend, table legend, main text, or Methods section.

n/a	Confirmed	
☐	☒	The exact sample size (*n*) for each experimental group/condition, given as a discrete number and unit of measurement
☐	☒	A statement on whether measurements were taken from distinct samples or whether the same sample was measured repeatedly
☐	☒	The statistical test(s) used AND whether they are one- or two-sided<br>*Only common tests should be described solely by name; describe more complex techniques in the Methods section.*
☒	☐	A description of all covariates tested
☐	☒	A description of any assumptions or corrections, such as tests of normality and adjustment for multiple comparisons
☐	☒	A full description of the statistical parameters including central tendency (e.g. means) or other basic estimates (e.g. regression coefficient) AND variation (e.g. standard deviation) or associated estimates of uncertainty (e.g. confidence intervals)
☒	☐	For null hypothesis testing, the test statistic (e.g. *F*, *t*, *r*) with confidence intervals, effect sizes, degrees of freedom and *P* value noted<br>*Give P values as exact values whenever suitable.*
☒	☐	For Bayesian analysis, information on the choice of priors and Markov chain Monte Carlo settings
☒	☐	For hierarchical and complex designs, identification of the appropriate level for tests and full reporting of outcomes
☒	☐	Estimates of effect sizes (e.g. Cohen's *d*, Pearson's *r*), indicating how they were calculated

Our web collection on statistics for biologists contains articles on many of the points above.

Software and code

Policy information about availability of computer code

Data collection	Chromeleon 7.2, ZetaView 8.05.05 SP2, NIS-Elements v. 5.00
Data analysis	Chromeleon 7.2, ZetaView 8.05.05 SP2, NIS-Elements v. 5.00 , MaxQuant v2.1.3.0, GraphPad Prism v. 8.0.0, Microsoft Office

For manuscripts utilizing custom algorithms or software that are central to the research but not yet described in published literature, software must be made available to editors and reviewers. We strongly encourage code deposition in a community repository (e.g. GitHub). See the Nature Portfolio guidelines for submitting code & software for further information.

Data

Policy information about availability of data

All manuscripts must include a data availability statement. This statement should provide the following information, where applicable:

- Accession codes, unique identifiers, or web links for publicly available datasets
- A description of any restrictions on data availability
- For clinical datasets or third party data, please ensure that the statement adheres to our policy

All data generated or analyzed during this study are included in this published article and its supplementary information files. Proteomics data are available as Supplementary Data 1 and via ProteomeXchange with identifier PXD037453. Raw data of Figures 2, 3 and 6 are available as Supplementary Data 2.

Human research participants

Policy information about studies involving human research participants and Sex and Gender in Research.

Reporting on sex and gender	Not applicable
Population characteristics	Not applicable
Recruitment	Not applicable
Ethics oversight	Not applicable

Note that full information on the approval of the study protocol must also be provided in the manuscript.

Field-specific reporting

Please select the one below that is the best fit for your research. If you are not sure, read the appropriate sections before making your selection.

☒ Life sciences ☐ Behavioural & social sciences ☐ Ecological, evolutionary & environmental sciences

For a reference copy of the document with all sections, see nature.com/documents/nr-reporting-summary-flat.pdf

Life sciences study design

All studies must disclose on these points even when the disclosure is negative.

Sample size	A sample size of at least three biologically independent replicates was chosen as it is considered the minimum in the field, while still manageable in terms of time and cost.
Data exclusions	No data was excluded. A system was in place, whereby with bigger sample sizes (at least n=9), an outlier test (Grubbs' test, also known as extreme studentized deviate (ESD) method) might have been performed, and datapoints could be excluded if proper scientific explanation could be additionally provided. However, this was never needed.
Replication	Experiments were performed at least 3 independent times (performed on different days, different passage numbers, sometimes different laboratories in different Countries).
Randomization	Not applicable. 5 out of the 6 treatment groups in the study are a type of control.
Blinding	Not applicable for this in vitro study. Although not relevant for this study, the staff at the Proteomics and Mass Spectrometry Core Facility (University of Bern, Switzerland) did not know what the treatments in the study were, only the authors' chosen labeling. The same is true for Dr. Chiara De Rossi (Helmholtz Institute for Pharmaceutical Research, Saarbrücken, Germany) and Dr. Jana Stamm (Friedrich Schiller University, Jena, Germany) during the acquisition of scanning electron microscopy images and cryogenic transmission electron microscopy images, respectively.

Reporting for specific materials, systems and methods

We require information from authors about some types of materials, experimental systems and methods used in many studies. Here, indicate whether each material, system or method listed is relevant to your study. If you are not sure if a list item applies to your research, read the appropriate section before selecting a response.

Materials & experimental systems

n/a	Involved in the study	
☐	☒	Antibodies
☐	☒	Eukaryotic cell lines
☒	☐	Palaeontology and archaeology
☒	☐	Animals and other organisms
☒	☐	Clinical data
☒	☐	Dual use research of concern

Methods

n/a	Involved in the study	
☒	☐	ChIP-seq
☐	☒	Flow cytometry
☒	☐	MRI-based neuroimaging

Antibodies

Antibodies used	Goat anti-Human IgG (H+L) Cross-Adsorbed Secondary Antibody (Alexa Fluor®488 conjugated and unconjugated) were from Thermo Fisher Scientific (Waltham, United States). Anti-Human CD81 Antibody (IgG2B, Alexa Fluor488-conjugated and unconjugated, Clone #454720), anti-Human CD9 (Alexa Fluor488-conjugated and unconjugated, IgG2b, Clone #209306), anti-human SPARC and GPC1 (polyclonal IgG, unconjugated), anti-human IDE (Mouse IgG1, AlexaFluor®488 conjugated, Clone #334501).
Validation	Validation was only performed in for the anti-human CD81 AlexaFluor488 conjugated antibody, the performance of which in our system was compared directly to its counterpart conjugated using the Lighting-Link® (LL) antibody labelling kit (Biotechne). The data for this is part of the manuscript. All antibodies, however, were chosen also because widely established in the literature for years.

Eukaryotic cell lines

Policy information about cell lines and Sex and Gender in Research

Cell line source(s)	LX-2 Human Hepatic Stellate Cell Line was purchased from Millipore Sigma (cat. nr. SCC064)
Authentication	Certificate of authenticity was provided upon purchase.
Mycoplasma contamination	Mycoplasma testing was routinely performed in all research laboratories at least once every 3 months for cells grown without antibiotics for at least 2 passages.
Commonly misidentified lines (See ICLAC register)	Not applicable.

Flow Cytometry

Plots

Confirm that:

- [x] The axis labels state the marker and fluorochrome used (e.g. CD4-FITC).
- [x] The axis scales are clearly visible. Include numbers along axes only for bottom left plot of group (a 'group' is an analysis of identical markers).
- [x] All plots are contour plots with outliers or pseudocolor plots.
- [x] A numerical value for number of cells or percentage (with statistics) is provided.

Methodology

Sample preparation	Not applicable
Instrument	BD LRS Fortessa (BD Biosciences)
Software	FACS Diva 8.0, FlowJo v10.8.1
Cell population abundance	Not applicable, flow cytometry was not performed on cells.
Gating strategy	Flow cytometry was not performed on cells, only on a homogeneous suspension of standard 5 um latex beads, with only one fluorophore. A representative figure can be provided upon request to show what signal was considered positive and what not.

- [ ] Tick this box to confirm that a figure exemplifying the gating strategy is provided in the Supplementary Information.
